# Persistent gaps in nutrition education in UK medical schools: a triangulated review of curricula, student perception and the evidence base

**DOI:** 10.1136/bmjnph-2025-001479

**Published:** 2026-04-20

**Authors:** Amanda Shiach, Abigail C Thomson, Fiona Samuels

**Affiliations:** 1 Centre for Public Health and Policy, Queen Mary University of London Wolfson Institute of Population Health, London, UK; 2 Queen Mary University of London Centre for Psychiatry and Mental Health, London, UK; 3 Wolfson Institute of Population Health, Queen Mary University of London, London, UK

**Keywords:** Dietary patterns, Malnutrition, Nutrition assessment, Preventive counselling, Nutritional treatment

## Abstract

**Background:**

Nutrition is a modifiable driver of morbidity and mortality in the UK, yet nutrition education remains marginal within medical curricula. Despite guidance from the General Medical Council (GMC) and the Association for Nutrition (AfN), evidence suggests persistent under-provision.

**Methods:**

We triangulated findings from: (1) a curriculum review and mapping exercise of UK medical schools; (2) a curriculum survey (n=31) of final-year or newly qualified students; and (3) a rapid review (2010–2025) of literature on nutrition education in UK medical programmes. Survey items mapped to AfN competencies and GMC Outcomes for Graduates.

**Results:**

Across all data sources, nutrition education was inconsistently delivered, theoretical and rarely assessed. Most students (71%) reported receiving ≤10 hours of teaching. Although 84% rated nutrition as important, only 61% felt prepared to address nutrition issues. Confidence was lowest for practical skills (nutrition assessment, referral pathways and brief interventions). The rapid review identified longstanding barriers: low curricular legitimacy, limited assessment, insufficient faculty expertise and weak clinical integration. Enablers included nutrition leads, interprofessional learning and competency-aligned teaching.

**Conclusions:**

Despite national prevention ambitions, nutrition remains insufficiently integrated into UK medical education. Aligning curricula with AfN/GMC standards, strengthening assessment, embedding leadership and co-designing applied teaching with students represent practical high-impact opportunities to develop prevention.

## Introduction

 During a lecture by the lead author on non-communicable diseases (NCDs) and the commercial determinants shaping UK food environments, only a handful of medical students attended. Although recorded, the apparently low engagement with a major clinical and public health issue—poor diet being a leading modifiable risk factor for premature mortality—was striking. This raised the core question motivating this study: if nutrition is not visibly learnt, assessed or institutionally valued, how can students appreciate its clinical and societal relevance? Low visibility in teaching often signals low importance to learners. Within curriculum theory, this is understood as ‘low curricular legitimacy’, in which topics lacking assessment, ownership or faculty expertise are perceived as peripheral. In the case of nutrition, this risks undermining the foundations of preventive care.

This brief report presents findings from a triangulated assessment of UK medical nutrition education using three data sources: curriculum mapping across UK medical schools; a survey of final-year or newly qualified students and a rapid review of literature since 2010. Together, these strands offer a system-level picture of how the current educational landscape aligns with national ambitions for prevention, workforce capability and improving clinical outcomes related to diet.

## Background

Diet-related disease is a major cause of premature mortality and disability in the UK.^1^ The National Health Service (NHS) Long Term Plan calls for a shift ‘from treatment to prevention’, recognising the influence of social, economic and commercial determinants on population health.^2^ Although the plan lacks a detailed nutrition strategy, it creates a policy window in which strengthening nutrition competencies becomes a credible lever for improving clinical care. With diet implicated in multiple long-term conditions, including cardiovascular disease, diabetes and obesity, the rationale for enhancing medical training is clear. Yet progress remains slow, and nutrition continues to be marginalised despite growing national attention to prevention.

Evidence consistently shows that nutrition education within UK medical curricula is limited. The realist review by Blythe *et al*.^3^ identifies low curricular legitimacy, insufficient expertise and lack of assessment as persistent barriers. Broad and Wallace^4^ similarly report that nutrition is often treated as peripheral rather than integral to training. A wider body of UK and international literature highlights fragmented provision and weak integration of biomedical, clinical and public health nutrition.^5–13^ Collectively, these studies point to longstanding structural issues requiring coordinated, system-level solutions.

## Methods

### Curriculum mapping

We systematically reviewed publicly available curriculum documentation from UK medical schools. Extraction captured visibility and location of nutrition content; alignment with Association for Nutrition (AfN) curriculum domains;^14^ applied clinical nutrition coverage; vertical integration across years; governance structures for nutrition oversight and interprofessional learning opportunities. Documents were included if they provided explicit, identifiable curriculum information and covered core undergraduate medical training (online supplemental file 1).

### Student survey (n=31)

A curriculum review survey was conducted with final-year or newly qualified medical students. Participation was voluntary, with 31 low-value vouchers distributed to respondents. Ethical approval was not required as this was a quality improvement activity. The survey assessed hours and visibility of nutrition teaching, perceived importance, preparedness and confidence across 11 AfN and General Medical Council (GMC) competencies, with additional open-text reflections. Competency items were derived directly from AfN and GMC^14 15^ frameworks to ensure relevance to clinical expectations (online supplemental file 2).

### Rapid review

A rapid review (2010–2025) included UK studies and international studies explicitly addressing UK medical nutrition education in higher education institutions. Data extraction captures study characteristics, study design, study findings and recommendations. Triangulation allowed comparison of formal curricular intentions, student experience and the academic evidence base, strengthening the overall interpretive validity (online supplemental file 3.

## Results

### Curriculum Mapping

Published nutrition content across UK medical school curricula was generally fragmented, inconsistently delivered and concentrated in early pre-clinical teaching. Most programmes included elements of biochemistry or physiology, yet substantive clinical nutrition—including malnutrition, obesity, frailty and micronutrient deficiencies—was largely absent. Key areas were rarely taught in an applied or clinically embedded way, echoing long-standing concerns about limited clinical integration.^2 3 5 6^ Structured teaching on dietary assessment or brief interventions was similarly restricted, and few programmes demonstrated clear alignment with AfN competencies^14^ or GMC expectations.^15^ Governance structures were minimal: most schools lacked a designated nutrition lead or mechanisms for coordinated oversight. While a small number of schools delivered more coherent and vertically integrated models—notably Brighton and Sussex Medical School, where compulsory, clinically oriented nutrition and lifestyle education is embedded across all years—these remained the exception rather than the norm. Markedly, universities offering AfN-accredited nutrition degrees and/or intercalated options were associated with greater nutrition teaching provision within the medical curriculum. Broader contextual teaching on social and commercial determinants of diet was also scarce, despite its relevance to UK prevention policy.^4^ Overall, these patterns mirror gaps identified across multiple UK studies^2 3 5 6^ and highlight persistent structural challenges, although emerging exemplars show that more integrated, practice-focused approaches are both feasible and effective (online supplemental file 4).

### Student survey

Students viewed nutrition as clinically important yet insufficiently taught or integrated across the MBBS programme. Most reported limited formal exposure: 22/31 (71%) had received 10 hours or fewer of nutrition teaching, whereas 25/31 (80%) believed at least 11 hours should be included. Over half (17/31, 55%) felt 16 hours or more would be appropriate. Several perceived a mismatch between the prominence of diet in clinical presentations—particularly multimorbidity, obesity and frailty—and its marginal curricular visibility (online supplemental file 5).

A clear divergence emerged between perceived importance and preparedness. Students rated the importance of nutrition education highly (mean 8.9/10; 84% scoring ≥8), yet their preparedness to identify, advise and refer for nutrition-related issues was substantially lower (mean 4.9/10; 61% scoring ≤5). This pattern reflects broader evidence linking competence to deliberate practice, structured assessment and clinical exposure.

Confidence across AfN-aligned competencies followed the same gradient. Students reported greater confidence with conceptual knowledge (eg, dietary principles, fluid and electrolyte balance) than with applied clinical skills. Only 9/31 (29%) felt capable of conducting a basic nutrition assessment, and 11/31 (36%) felt safe referencing nutrient requirements or recognising deficiencies. Just over half (17/31, 55%) felt able to refer appropriately to dietitians or specialist services. ‘Very safe’ ratings were uncommon across all competencies.

Clinical observation opportunities were also limited: only 4/31 (13%) had routinely seen clinicians initiate nutrition-focused conversations, and very few had worked alongside dietitians or other nutrition professionals. Open-text responses described nutrition as ‘assumed knowledge’ and ‘everywhere in theory but nowhere in exams’, with strong calls for more applied teaching, clearer referral pathways and better integration of socio-economic and cultural perspectives.

### Rapid review findings

The 12 identified studies included surveys, curriculum evaluations, qualitative focus groups and consensus-building approaches (eg, Delphi), providing a diverse evidence base. Consistent structural and pedagogical barriers were identified. Several papers highlighted the low curricular legitimacy of nutrition compared with high-status biomedical subjects.^3–5^ This was compounded by insufficient faculty expertise^5 6^ and the lack of meaningful assessment, a factor associated with weak student engagement.^3–7^ Across curriculum evaluations and scoping reviews, nutrition teaching was characterised as fragmented, predominantly theoretical and inconsistently delivered.^8 9^ Clinical integration was limited, and opportunities for interprofessional learning with dietitians and other nutrition professionals were scarce.^3–6^ Alignment with competency frameworks (including AfN and Delphi-derived consensus^9^) was variable and often weak (online supplemental file 6).^6–12^

Alongside these barriers, several enablers were identified. Appointing a designated Nutrition Lead improved coordination and visibility.^2^ Integrated applied clinical teaching strengthened competence^5 6^, while competency-aligned curricula—including AfN and Delphi frameworks—supported clearer learning expectations.^8–11 14^ Interprofessional learning with dietitians and other nutrition professionals enhanced skills and confidence,^3^ and embedding nutrition in high-stakes assessment increased curricular legitimacy.^7 9^ Evidence from evaluations of nutrition education interventions, such as case-based workshops, supports the value of applied, experiential teaching.^13^ See table 1.

**Table 1:** Summary of triangulated findings

Domain	Curriculum mapping	Student survey	Rapid review	Implications
Teaching exposure	Fragmented, theoretical	71%≤10 hours	Persistent under-provision	Increase dedicated, applied time
Assessment	Rare	‘Nowhere in exams’	Critical barrier	Incorporate in existing assessments*
Applied skills	Limited	Low confidence	Key deficit	Case-based/clinical teaching**
Leadership	Few nutrition leads	n/a	Enabler	Appoint leads
Determinants of diet	Minimal content	Students request more	Under-taught	Integrate commercial determinants of health
Curriculum design and responsiveness	No systematic mechanism for student input	Demand for applied practical reform	Benefits of learner-informed curriculum development	Embed co-production in curriculum review cycles

*Single best answer (SBA) and observed structural clinical examinations (OSCE).

**Multidisciplinary Team (MDT) with their range of expertise.

## Interpretation

Findings across all strands show that nutrition education in most UK medical schools remains insufficient, inconsistently delivered and weakly assessed.^2 3 5 6 8 9 12^Students recognise its clinical importance but feel under-prepared to deliver nutrition-related care (survey data; online supplemental file 5). Given the UK’s heavy burden of diet-related disease^1^, this represents a missed opportunity for prevention, particularly as addressing dietary risk factors is central to managing multimorbidity and improving clinical outcomes.

Nutrition’s low curricular status may reflect broader cultural dynamics. As most UK dietitians and school food educators are women, nutrition is sometimes framed as ‘soft’, contributing to curricular marginalisation. Though beyond this study’s scope, this lens^15^ may explain persistent institutional deprioritisation. Competency analyses also highlight structural barriers to embedding nutrition in medical training.^10 11^

The newly implemented UK Medical Licensing Assessment includes several nutrition-linked competencies aligned with GMC outcomes for graduates.^16^ Without strengthened teaching and assessment, graduates may be under-prepared for both licensure and routine clinical practice.

## Policy and practice implications

**Figure 1:**
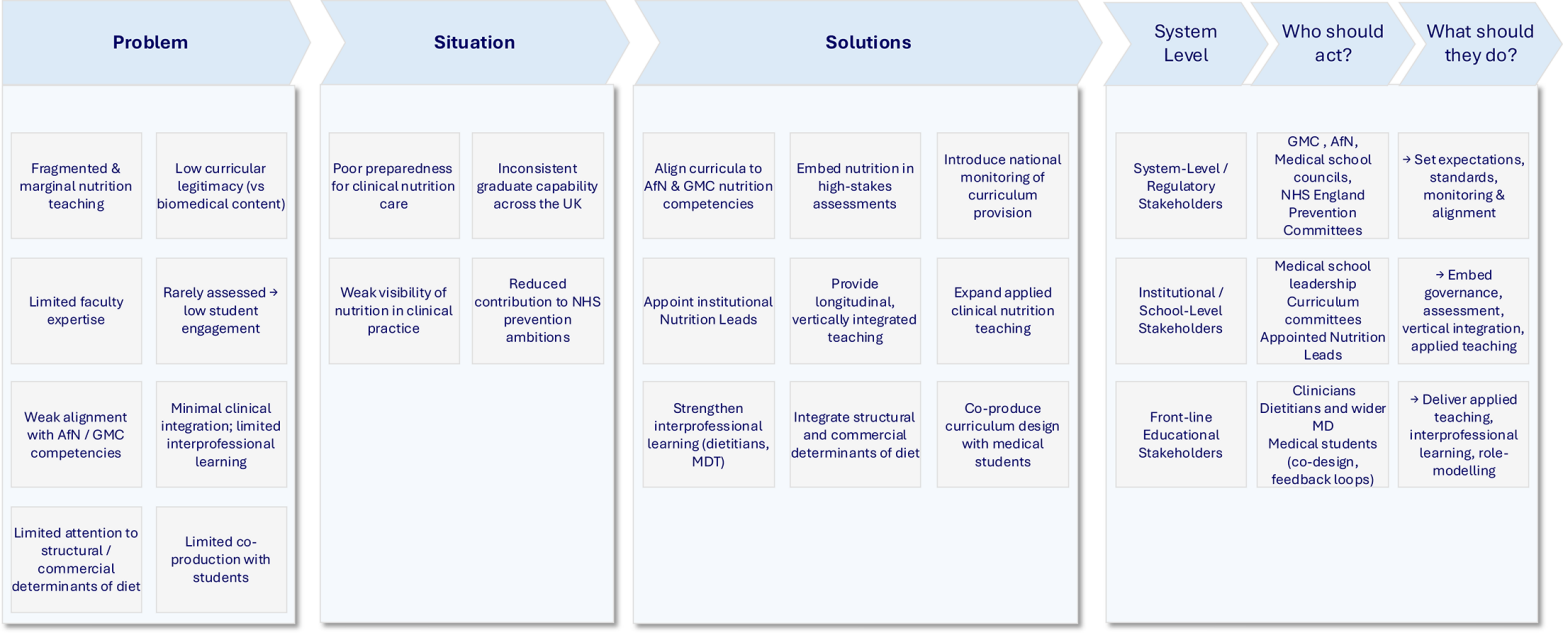
System-level barriers and solutions for strengthening nutrition education in UK medical education. AfN, Association for Nutrition; GMC, General Medical Council; MDT, multidisciplinary team; NHS, National Health Service.

Strengthening medical nutrition education requires coordinated system-level action. Priorities include aligning curricula with AfN and GMC competencies, ^14 15^embedding nutrition in high-stakes assessments, ^10–12^appointing institutional Nutrition Leads^2^and expanding applied clinical and interprofessional teaching.^3 5 6^ Integrating commercial and structural determinants of diet^⁴^ and establishing national monitoring of provision^12^would raise curricular legitimacy, while co-producing teaching with students enhances relevance and uptake. Together, these steps provide practical levers to deliver the NHS prevention ambitions^2^ (see figure 1).

## Conclusion

Nutrition education in most UK medical schools remains insufficient, inconsistently delivered and weakly assessed. With diet-related disease placing exceptional pressure on the NHS, strengthening nutrition training represents an immediate, high-leverage opportunity to enhance preventive care. Implementing AfN- and GMC-aligned, clinically applied nutrition teaching would better prepare future doctors to bridge individual care and public health, support management of the UK’s NCD burden and advance national prevention goals.

## Supplementary material

10.1136/bmjnph-2025-001479 online supplemental file 1

10.1136/bmjnph-2025-001479 online supplemental file 2

10.1136/bmjnph-2025-001479 online supplemental file 3

10.1136/bmjnph-2025-001479 online supplemental file 4

10.1136/bmjnph-2025-001479 online supplemental file 5

10.1136/bmjnph-2025-001479 online supplemental file 6
