# Supplementary material for: Persistent gaps in nutrition education in UK medical schools: a triangulated review of curricula, student perception and the evidence base
Source: BMJ Nutr Prev Health. 2026 Apr 20;9(1):e001479. doi: 10.1136/bmjnph-2025-001479 (PMC13425111; doi:10.1136/bmjnph-2025-001479)
Supplement: online supplemental file 1 [file bmjnph-9-1-s001.pdf]

Eligibility Criteria for Rapid Review

Inclusion

1. UK-based medical schools delivering training to medical students with a nutrition course/module/content (Scotland, Wales, England, Northern Ireland)
2. Can be delivered in any type of institution where you could apply as a UK student to do medicine

Exclusion

1. Non-UK-based medical schools or institutions
2. No teaching delivered on nutrition
3. Insufficient data on course modules or teaching to understand what has been delivered

Data to Extract

The following information will be sought from each source:

- **Institution Characteristics**
  - Name of institution
  - Region of the UK
  - League table statistics
- **Student Characteristics**
  - Number of students enrolled in this programme
  - Demographic characteristics of enrolled students
    - Gender
    - Ethnicity
  - Number of students enrolled in the nutrition course (if optional)
  - % of overall enrolled students
  - Demographic characteristics of enrolled students
    - Gender
    - Ethnicity
- **Nutrition Content**
  - Who? (i.e. internal or external facilitation, community providers)
  - Compulsory or optional
  - When delivered (i.e. which year)
  - Method of delivery (i.e. lecture, practicals, visits)
  - Frequency (i.e. one-off, multiple sessions)
  - Length (i.e. duration of sessions)
  - Details about the content delivered (focus, topics addressed)
  - Is this course evaluated (Yes/No)
    - If so, how? (describe)
  - Is this course accredited?

Possible Institutions to Review

England

- Anglia Ruskin University School of Medicine
- Aston University Medical School

- Brighton & Sussex Medical School
- Bristol Medical School
- Brunel Medical School
- Durham University School of Medicine and Health
- Edge Hill University Medical School
- Hull York Medical School
- Imperial College School of Medicine
- Keele University School of Medicine
- King's College London GKT
- Lancaster Medical School
- Leeds School of Medicine
- Leicester Medical School
- Lincoln Medical School (UG pre-clinical + clinical elsewhere)
- Liverpool Medical School
- Manchester Medical School
- Newcastle University Medical School
- Norwich Medical School (UEA)
- Oxford Medical Sciences Division
- Peninsula Medical School (Plymouth)
- St George's, University of London
- Sheffield Medical School
- Southampton Medical School
- UCL Medical School
- University of Buckingham Medical School
- University of Central Lancashire School of Medicine
- University of Chester Medical School (graduate-entry)
- University of Exeter Medical School
- University of Nottingham Medical School (Nottingham & Lincoln – graduate-entry)
- University of Sunderland School of Medicine
- Three Counties Medical School (Worcester) (graduate-entry)
- Warwick Medical School

Scotland

- University of Aberdeen School of Medicine
- University of St Andrews School of Medicine
- University of Dundee Medical School
- University of Edinburgh Medical School
- University of Glasgow Medical School

Wales

- Cardiff University School of Medicine
- Swansea University Medical School
- North Wales Medical School (Bangor)

Northern Ireland

- Queen's University Belfast Medical School
- Ulster University School of Medicine (Magee, Derry)
