# Supplementary material for: Persistent gaps in nutrition education in UK medical schools: a triangulated review of curricula, student perception and the evidence base
Source: BMJ Nutr Prev Health. 2026 Apr 20;9(1):e001479. doi: 10.1136/bmjnph-2025-001479 (PMC13425111; doi:10.1136/bmjnph-2025-001479)
Supplement: online supplemental file 2 [file bmjnph-9-1-s002.pdf]

Rapid Review of Undergraduate Curriculum in Nutrition for Medical Doctors at QMUL.

Poor diet is the leading modifiable risk factor for ill health in the UK, contributing to the burden of obesity, type 2 diabetes, cardiovascular disease, and certain cancers.[1] Given this, doctors are expected to play a key role in preventive health and dietary counselling. However, most UK medical graduates report feeling underprepared to deliver effective nutrition advice. We are keen to understand your experience of nutrition training within the foundational medical education at QMUL, and if you feel adequately prepared to consider nutrition factors within assessments of patients' presentation and care. **REF**. *[1] Fadnes LT, Økland J-M, Haaland ØA, Johansson KA (2022) Estimating impact of food choices on life expectancy: A modeling study. PLoS Med 19(2): e1003889. https://doi.org/10.1371/journal.pmed.1003889*

* Required

1

Please answer the survey questions thoroughly to contribute meaningfully to this review. Tick below to indicate if you would like to know the outcome of this rapid review and the report will be emailed to you (your email is collected automatically). *

- ◯ Yes
- ◯ No
- ◯ Option 3

2

On completion, you will be sent a £10 voucher. The survey will be closed once all vouchers have been administered. Please supply your email address below to receive a digital voucher in early September. (NB. this is a change from earlier recipents who received their vouchers by post.) *

3

The statements below are taken from the Association for Nutrition undergraduate curriculum for nutrition *[2] [AfN UK Undergraduate Curriculum in Nutrition for Medical Doctors, © AfN 2021].* Based on your MBBS nutrition education, how safe and confident would you feel to fulfil the criteria listed below: *

	Very safe	Somewhat safe	Neither safe nor unsafe	Somewhat unsafe	Very unsafe
1. Detect patients who are underweight or at risk of undernutrition and patients who are living with excess weight.	○	○	○	○	○
2. Include a basic assessment of nutritional status into every patient's clerking and state, where appropriate, a nutritional care plan/referral.	○	○	○	○	○
3. Know the importance of fluid and electrolyte requirements in health and during illness (including post-operatively).	○	○	○	○	○
4. Describe how giving nutritional support to undernourished patients and achieving movement towards a healthy weight in individuals who are underweight/overweight or with obesity can improve quality of life, morbidity and/or mortality.	○	○	○	○	○
5. Describe the importance of diet in maintaining health in all ages, life-stages, sex and ethnic groups.	○	○	○	○	○

	Very safe	Somewhat safe	Neither safe nor unsafe	Somewhat unsafe	Very unsafe
6. Undertake a basic assessment of nutritional and hydration status to identify undernutrition, its consequences and treatment/ management.	○	○	○	○	○
7. Identify individuals who are living with excess weight at all ages and the related health risk consequences and be aware of the treatment/management options, appreciating micronutrient deficiency may also be a factor to consider.	○	○	○	○	○
8. Have an understanding of, and know where to reference, the approximate average energy, macronutrient, water, micronutrient requirements and the more common deficiencies in the UK population.	○	○	○	○	○
9. Describe the official UK population dietary recommendations and be able to promote a "healthy balanced diet".	○	○	○	○	○
10. Describe the indications for patients needing clinically assisted nutrition and hydration support (oral, enteral and parenteral); know the principles of administration and the potential complications.	○	○	○	○	○

	Very safe	Somewhat safe	Neither safe nor unsafe	Somewhat unsafe	Very unsafe
11. Understand when it is appropriate to refer for specialist nutrition/dietetic support and/or to specialist nutrition/weight management services.	○	○	○	○	○

4

The statements below are taken from the Association for Nutrition undergraduate curriculum for nutrition *[2] [AfN UK Undergraduate Curriculum in Nutrition for Medical Doctors, © AfN 2021]*. Based on your MBBS nutrition education, how safe and confident would you feel to fulfil the criteria listed below: *

	Very safe	Somewhat safe	Neither safe nor unsafe	Somewhat unsafe	Very unsafe
1. Detect patients who are underweight or at risk of undernutrition and patients who are living with excess weight.	◯	◯	◯	◯	◯
2. Include a basic assessment of nutritional status into every patient's clerking and state, where appropriate, a nutritional care plan/referral.	◯	◯	◯	◯	◯
3. Know the importance of fluid and electrolyte requirements in health and during illness (including post-operatively).	◯	◯	◯	◯	◯
4. Describe how giving nutritional support to undernourished patients and achieving movement towards a healthy weight in individuals who are underweight/overweight or with obesity can improve quality of life, morbidity and/or mortality.	◯	◯	◯	◯	◯
5. Describe the importance of diet in maintaining health in all ages, life-stages, sex and ethnic groups.	◯	◯	◯	◯	◯

	Very safe	Somewhat safe	Neither safe nor unsafe	Somewhat unsafe	Very unsafe
6. Undertake a basic assessment of nutritional and hydration status to identify undernutrition, its consequences and treatment/ management.	◯	◯	◯	◯	◯
7. Identify individuals who are living with excess weight at all ages and the related health risk consequences and be aware of the treatment/management options, appreciating micronutrient deficiency may also be a factor to consider.	◯	◯	◯	◯	◯
8. Have an understanding of, and know where to reference, the approximate average energy, macronutrient, water, micronutrient requirements and the more common deficiencies in the UK population.	◯	◯	◯	◯	◯
9. Describe the official UK population dietary recommendations and be able to promote a “healthy balanced diet”.	◯	◯	◯	◯	◯
10. Describe the indications for patients needing clinically assisted nutrition and hydration support (oral, enteral and parenteral); know the principles of administration and the potential complications.	◯	◯	◯	◯	◯

	Very safe	Somewhat safe	Neither safe nor unsafe	Somewhat unsafe	Very unsafe
11. Understand when it is appropriate to refer for specialist nutrition/dietetic support and/or to specialist nutrition/weight management services.	◯	◯	◯	◯	◯

5

How many hours do you think you have spent on nutrition education during the whole MBBS? Throughout the course, you will have received lectures on **biochemistry** e.g. carbohydrate metabolism: the digestion and absorption of carbohydrates, focusing on insulin and glucagon's roles in regulating blood glucose levels and **clinical training** to understand nutritional needs across the lifespan, recognise the impact of diet on health and disease, and learned how to apply dietary principles in patient care. *

- ◯ 5 hours or less
- ◯ 6-10 hours
- ◯ 11-15 hours
- ◯ 16-20 hours
- ◯ 21-25 hours
- ◯ 26+ hours

6

What do you think is the ideal time to spend on nutrition education during the whole MBBS? *

- ◯ 5 hours or less
- ◯ 6-10 hours
- ◯ 11-15 hours
- ◯ 16-20 hours
- ◯ 21-25 hours
- ◯ 26+ hours

7

Thinking back over the nutrition education you received as an MBBS student, please highlight key experiences you recall. These may have been PBL scenarios, small group activities, online modules, lectures or clinical-based experience e.g., in diabetes related services. Include the lecturer, year, or module, for example, specific Y3 Public Health lectures. *

8

What further nutrition content would you suggest be included in the MBBS at QMUL? Please suggest specific topics; e.g., dietary advice, illnesses related to malnutrition, socio-economic insights to dietary preference, skills; e.g., cooking skills, dietary history assessment, and teaching style; e.g., workshops, lectures, e-learning, ward-based, student-selected modules.
*

9

How far would you agree that nutrition education is important in medical school training? *

0	1	2	3	4	5	6	7	8	9	10

Strongly disagree — Completely agree

10

Overall, how well prepared do you feel to identify, advise, and refer patients for nutrition-related issues and illnesses? *

0	1	2	3	4	5	6	7	8	9	10

Not at all prepared — Extremely well prepared

11

GMC Outcomes for graduates: Safeguarding vulnerable patients
7. Newly qualified doctors must be able to recognise and identify factors that suggest patient vulnerability and take action in response. **Are you able to recognise where** addiction (to drugs, alcohol, smoking or other substances), **poor nutrition**, self neglect, environmental exposure, or financial or social deprivation are *[is}* **contributing to ill health. And take action by seeking advice from colleagues and making appropriate referrals?** *

	Yes, absolutely	Most of the time	Probably sufficiently	Little of the time	No, not at all
Statement 1	◯	◯	◯	◯	◯

12

GMC Outcomes for graduates: Applying biomedical scientific principles
22. Newly qualified doctors must be able to apply biomedical scientific principles, methods and knowledge to medical practice and integrate these into patient care. **This must include principles and knowledge relating to nutrition. Based on your MBBS nutrition education how safe and confident would you feel to fulfil the criteria listed below:** *

	Very safe	Somewhat safe	Neither safe nor unsafe	Somewhat unsafe	Very unsafe
A. Explain how normal human structure and function and physiological processes applies, including at the extremes of age, in children and young people and during pregnancy and childbirth.	◯	◯	◯	◯	◯
B. Explain the relevant scientific processes underlying common and important disease processes.	◯	◯	◯	◯	◯
C. Justify, through an explanation of the underlying fundamental principles and clinical reasoning, the selection of appropriate investigations for common clinical conditions and diseases.	◯	◯	◯	◯	◯
D. Select appropriate forms of management for common diseases, and ways of preventing common diseas es, and explain their modes of action and their risks from first principles.	◯	◯	◯	◯	◯

	Very safe	Somewhat safe	Neither safe nor unsafe	Somewhat unsafe	Very unsafe
E. Describe medications and medication actions: therapeutics and pharmacokinetics; medication side effects and interactions, including for multiple treatments, long term physical and mental conditions and non-prescribed drugs; the role of pharmacogenomics and antimicrobial stewardship.	◯	◯	◯	◯	◯
F. Analyse clinical phenomena and conduct appropriate critical appraisal and analysis of clinical data, and explain clinical reasoning in action and how they formulate a differential diagnosis and management plan.	◯	◯	◯	◯	◯

13

GMC Outcomes for graduates: Health promotion and illness prevention. 25. Newly qualified doctors must be able to **apply the principles, methods and knowledge** of **population health** and the **improvement** of **health** and sustainable healthcare to medical practice. **Based on your MBBS nutrition education how safe and confident would you feel to fulfil the criteria listed below as relates to nutrition and health:** *

	Very safe	Somewhat safe	Neither safe nor unsafe	Somewhat unsafe	Very unsafe
A. Explain the concept of wellness or wellbeing as well as illness, and be able to help and empower people to achieve the best health possible, including promoting lifestyle changes such as smoking cessation, avoiding substance misuse and maintaining a healthy weight through physical activity and diet.	○	○	○	○	○
B. Describe the health of a population using basic epidemiological techniques and measurements.	○	○	○	○	○
C. Evaluate the environmental, social, behavioural and cultural factors which infuence health and disease in different populations.	○	○	○	○	○
D. Assess, by taking a history, the environmental, social, psychological, behavioural and cultural factors influencing a patient's presentation, and identify options to address these, including advocacy for those who are disempowered	○	○	○	○	○
**E. Discuss the role and impact of nutrition to the health of individual patients and societies**	○	○	○	○	○

	Very safe	Somewhat safe	Neither safe nor unsafe	Somewhat unsafe	Very unsafe
F. Evaluate the determinants of health and disease and variations in healthcare delivery and medical practice from a global perspective and explain the impact that global changes may have on local health and wellbeing.	○	○	○	○	○

14

How far would you agree that poor diet is the number one killer of people in the UK? *

0	1	2	3	4	5	6	7	8	9	10

Not agree at all — Strongly agree

15

This 5 minute video from the US presents a particular view on nutrition training for doctors, based on selected academic papers. Please watch it and make any comments on ideas presented in the text box below. *

How Much Do Doctors Know About Nutrition?

16

Having watched the video, how far do you agree with the message that there is insufficient emphasis on nutrition in medical training generally? *

0	1	2	3	4	5	6	7	8	9	10

Not agree at all — Strongly agree

17

These organisations promote the teaching of nutrition for doctors. Please tick any have you heard of. *

- [ ] Association for Nutrition (AfN) Undergraduate Curriculum in Nutrition for Medical Doctors
- [ ] Nutritank
- [ ] NNEdPro Global Institute for Food, Nutrition and Health
- [ ] Culinary Medicine UK
- [ ] Education and Research In Medical Nutrition Network (ERIMNN)
- [ ] The Nutrition Implementation Coalition
- [ ] None of the above

This content is neither created nor endorsed by Microsoft. The data you submit will be sent to the form owner.

Microsoft Forms
