# Supplementary material for: Persistent gaps in nutrition education in UK medical schools: a triangulated review of curricula, student perception and the evidence base
Source: BMJ Nutr Prev Health. 2026 Apr 20;9(1):e001479. doi: 10.1136/bmjnph-2025-001479 (PMC13425111; doi:10.1136/bmjnph-2025-001479)
Supplement: online supplemental file 3 [file bmjnph-9-1-s003.pdf]

MethodsEligibility Criteria

Inclusion

1. Any paper that reviews, discusses or evaluates the inclusion of a nutrition course/module/content within any type of medical school in the UK (England, Wales. Scotland, Ireland)
2. Published from 2010 to 15/10/2025
3. Any study design, including reviews and commentaries
4. Published in English

Exclusion

1. Papers that do not include any data or discussion of content relevant to the UK-based medical school curriculum
2. Broader medical curriculum evaluations that do not discuss nutrition content specifically

Search Strategy Web of Science

#1 - TS=(evaluat* OR assess* OR "learning outcome*" OR "educational outcome*" OR "curriculum outcome*" OR effective* OR impact OR impacts OR efficacy OR "program evaluation" OR "training evaluation" OR "educational assessment" OR feedback OR "knowledge gain" OR "skills assessment" OR "competency assessment" OR implement* OR deliver* OR "program delivery" OR "curriculum delivery" OR integrate* OR adoption OR adopt* OR rollout OR execute* OR uptake OR incorporate* OR embedding OR embedded OR review OR commentary OR opinion)

#2 - TS=("medical education" OR "medical training" OR "undergraduate medical education" OR "graduate medical education" OR "medical school*" OR "medical student*" OR "medical curriculum" OR "medical curricula" OR "health professions education" OR "clinical education" OR "clinical training" OR "preclinical education" OR "preclinical training" OR "medical teaching" OR "medical instruction")

#3 - TS=(nutrition OR "nutrition education" OR "dietary education" OR "nutrition knowledge" OR "dietary knowledge" OR "nutrition awareness" OR "nutrition training" OR "nutrition counselling" OR "nutrition counseling" OR "nutrition instruction" OR "dietary counselling" OR "dietary counseling" OR "nutrition promotion" OR "food education" OR "healthy eating education" OR "nutrition literacy" OR "dietary literacy")

#1 AND #2 AND #3 and NORTH IRELAND or ENGLAND or SCOTLAND or WALES (Countries/Regions)
