# Supplementary material for: Persistent gaps in nutrition education in UK medical schools: a triangulated review of curricula, student perception and the evidence base
Source: BMJ Nutr Prev Health. 2026 Apr 20;9(1):e001479. doi: 10.1136/bmjnph-2025-001479 (PMC13425111; doi:10.1136/bmjnph-2025-001479)
Supplement: online supplemental file 5 [file bmjnph-9-1-s005.pdf]

Rapid Review of Undergraduate Curriculum in Nutrition for Medical Doctors at QMUL

Poor diet is the leading modifiable risk factor for ill health in the UK, contributing to the burden of obesity, type 2 diabetes, cardiovascular disease, and certain cancers.[1] Given this, doctors are expected to play a key role in preventive health and dietary counselling. However, most UK medical graduates report feeling underprepared to deliver effective nutrition advice. We are keen to understand your experience of nutrition training within the foundational medical education at QMUL, and if you feel adequately prepared to consider nutrition factors within assessments of patients' presentation and care. REF. [1] Fadnes LT, Økland J-M, Haaland ØA, Johansson KA (2022) Estimating impact of food choices on life expectancy: A modeling study. PLoS Med 19(2): e1003889. https://doi.org/10.1371/journal.pmed.1003889

Please answer the survey questions thoroughly to contribute meaningfully to this review. Tick below to indicate if you would like to know the outcome of this rapid review and the report will be emailed to you (your email is collected automatically).

Please contact the lead author if interested in the full text of any truncated comments. a.shiach@qmul.ac.uk

Questions \ Response ID	1	2	3	4	5	6	7	8	9	10	11	12	13	14	15	16
The statements below are taken from the Association for Nutrition undergraduate curriculum for nutrition [2] [AfN UK Undergraduate Curriculum in Nutrition for Medical Doctors, © AfN 2021]. Based on your MBBS nutrition education, how safe and confident would you feel to fulfil the criteria listed below:																
1. Detect patients who are underweight or at risk of undernutrition and patients who are living with excess weight.	Very safe	Very safe	Somewhat safe	Somewhat safe	Neither safe nor unsafe	Somewhat unsafe	Neither safe nor unsafe	Very safe	Somewhat safe	Somewhat unsafe	Somewhat unsafe	Somewhat safe	Very safe	Neither safe nor unsafe	Somewhat safe	Somewhat safe
2. Include a basic assessment of nutritional status into every patient's clerking and state, where appropriate, a nutritional care plan/referral.	Somewhat safe	Somewhat safe	Somewhat safe	Somewhat unsafe	Very unsafe	Somewhat unsafe	Somewhat unsafe	Somewhat unsafe	Somewhat unsafe	Very unsafe	Very unsafe	Somewhat unsafe	Very unsafe	Neither safe nor unsafe	Somewhat safe	Neither safe nor unsafe
3. Know the importance of fluid and electrolyte requirements in health and during illness (including post-operatively).	Very safe	Somewhat safe	Neither safe nor unsafe	Very safe	Somewhat safe	Somewhat safe	Somewhat safe	Very safe	Somewhat safe	Somewhat safe	Somewhat safe	Somewhat safe	Neither safe nor unsafe	Somewhat safe	Somewhat safe	Somewhat safe
4. Describe how giving nutritional support to undernourished patients and achieving movement towards a healthy weight in individuals who are underweight/overweight or with obesity can improve qual...	Somewhat safe	Very safe	Neither safe nor unsafe	Somewhat safe	Very unsafe	Somewhat safe	Somewhat unsafe	Somewhat safe	Very safe	Somewhat safe	Somewhat safe	Somewhat safe	Very unsafe	Somewhat safe	Somewhat unsafe	Somewhat safe
5. Describe the importance of diet in maintaining health in all ages, life-stages, sex and ethnic groups.	Very safe	Very safe	Somewhat safe	Very safe	Somewhat safe	Somewhat safe	Neither safe nor unsafe	Somewhat safe	Neither safe nor unsafe	Somewhat safe	Somewhat unsafe	Somewhat unsafe	Very unsafe	Somewhat safe	Very safe	Somewhat safe
6. Undertake a basic assessment of nutritional and hydration status to identify undernutrition, its consequences and treatment/ management.	Somewhat safe	Somewhat safe	Somewhat safe	Somewhat safe	Neither safe nor unsafe	Somewhat safe	Somewhat unsafe	Neither safe nor unsafe	Somewhat safe	Somewhat unsafe	Somewhat safe	Somewhat safe	Somewhat unsafe	Neither safe nor unsafe	Somewhat safe	Very safe
7. Identify individuals who are living with excess weight at all ages and the related health risk consequences and be aware of the treatment/management options, appreciating micronutrient deficien...	Very safe	Very safe	Somewhat safe	Somewhat safe	Very unsafe	Neither safe nor unsafe	Somewhat safe	Somewhat safe	Somewhat safe	Neither safe nor unsafe	Somewhat safe	Somewhat safe	Somewhat unsafe	Somewhat safe	Somewhat safe	Somewhat safe
8. Have an understanding of, and know where to reference, the approximate average energy, macronutrient, water, micronutrient requirements and the more common deficiencies in the UK population	Very safe	Somewhat safe	Neither safe nor unsafe	Somewhat unsafe	Somewhat safe	Somewhat unsafe	Neither safe nor unsafe	Neither safe nor unsafe	Somewhat safe	Somewhat safe	Somewhat unsafe	Somewhat safe	Very unsafe	Somewhat safe	Somewhat safe	Very safe
9. Describe the official UK population dietary recommendations and be able to promote a "healthy balanced diet".	Very safe	Somewhat unsafe	Somewhat safe	Neither safe nor unsafe	Very unsafe	Somewhat safe	Somewhat safe	Very safe	Very safe	Somewhat safe	Somewhat unsafe	Neither safe nor unsafe	Very unsafe	Somewhat safe	Somewhat safe	Very safe
10. Describe the indications for patients needing clinically assisted nutrition and hydration support (oral, enteral and parenteral); know the principles of administration and the potential compli...	Somewhat safe	Very safe	Somewhat safe	Somewhat safe	Neither safe nor unsafe	Neither safe nor unsafe	Neither safe nor unsafe	Somewhat unsafe	Neither safe nor unsafe	Neither safe nor unsafe	Somewhat unsafe	Somewhat safe	Very unsafe	Somewhat safe	Neither safe nor unsafe	Somewhat safe
11. Understand when it is appropriate to refer for specialist nutrition/dietetic support and/or to specialist nutrition/weight management services.	Somewhat safe	Somewhat unsafe	Somewhat safe	Neither safe nor unsafe	Very unsafe	Somewhat safe	Somewhat safe	Very safe	Somewhat safe	Somewhat safe	Very unsafe	Somewhat safe	Very unsafe	Somewhat safe	Neither safe nor unsafe	Somewhat safe
How many hours do you think you have spent on nutrition education during the whole MBBS? Throughout the course, you will have received lectures on biochemistry e.g. carbohydrate metabolism: the di...	21-25 hours	6-10 hours	5 hours or less	11-15 hours	5 hours or less	5 hours or less	6-10 hours	11-15 hours	11-15 hours	6-10 hours	6-10 hours	5 hours or less	5 hours or less	6-10 hours	6-10 hours	26+ hours
What do you think is the ideal time to spend on nutrition education during the whole MBBS?	26+ hours	21-25 hours	11-15 hours	26+ hours	16-20 hours	6-10 hours	11-15 hours	16-20 hours	21-25 hours	11-15 hours	26+ hours	16-20 hours	26+ hours	11-15 hours	16-20 hours	26+ hours
Thinking back over the nutrition education you received as an MBBS student, please highlight key experiences you recall. These may have been PBL scenarios, small group activities, online modules, ...	Y3 Nutrition Lectures Y3 Metabolism PBLs Y3 Gastroenterology lectures Diet in people from [illegible]	Sims of discussing weight. Discussing weight and diet in St Andrews as a DCE. Barts y3-5 placed little emphasis	PBL sessions in y1	I'm struggling to. I think conditions like refeeeing syndrome were stressed. There was also a lot of [illegible]	GP placement - consultations in which diet is discussed	I can't recall much apart from Public Health y2	Don't have any key experiences	None are memorable. More feeding teaching needed	I remember at some point learning about MUST assessments but I can't really remember what I learned. I [illegible]	((Joined in clinical years)) Lecture on prescribing exercise - included diet - Year 4 Public health lectures - re [illegible]	Many times we would say "give diet and lifestyle advice" without knowing what this means or going into [illegible]	Nutrition online module that had refeeding sybdrome in it. That seemed to be the most clinically relevant [illegible]	None to highlight	Placement in hospital for diabetes, public health lectures	I remember some lectures in metabolism modules and looking at nutrition in relation to different conditions.	In first year/second year there were PBLs on patients with PKU and the specialised diet they need. Also lectures on [illegible]

Questions \ Response ID	17	18	19	20	21	22	23	24	25	26	27	28	29	30	31
The statements below are taken from the Association for Nutrition undergraduate curriculum for nutrition [2] [AfN UK Undergraduate Curriculum in Nutrition for Medical Doctors, © AfN 2021]. Based on your MBBS nutrition education, how safe and confident would you feel to fulfil the criteria listed below:															
1. Detect patients who are underweight or at risk of undernutrition and patients who are living with excess weight.	Somewhat safe	Somewhat unsafe	Neither safe nor unsafe	Neither safe nor unsafe	Somewhat safe	Somewhat safe	Somewhat safe	Somewhat safe	Somewhat unsafe	Somewhat safe	Somewhat safe	Somewhat safe	Somewhat safe	Somewhat unsafe	Somewhat safe
2. Include a basic assessment of nutritional status into every patient's clerking and state, where appropriate, a nutritional care plan/referral.	Neither safe nor unsafe	Neither safe nor unsafe	Neither safe nor unsafe	Somewhat unsafe	Somewhat unsafe	Somewhat safe	Somewhat unsafe	Somewhat safe	Somewhat safe	Neither safe nor unsafe	Somewhat safe	Somewhat safe	Neither safe nor unsafe	Somewhat unsafe	Somewhat unsafe
3. Know the importance of fluid and electrolyte requirements in health and during illness (including post-operatively).	Somewhat safe	Somewhat safe	Neither safe nor unsafe	Somewhat safe	Very safe	Somewhat safe	Somewhat safe	Somewhat unsafe	Somewhat safe	Somewhat safe	Somewhat safe	Somewhat safe	Somewhat safe	Somewhat safe	Somewhat unsafe
4. Describe how giving nutritional support to undernourished patients and achieving movement towards a healthy weight in individuals who are underweight/overweight or with obesity can improve qual...	Somewhat safe	Neither safe nor unsafe	Somewhat safe	Neither safe nor unsafe	Very safe	Neither safe nor unsafe	Somewhat safe	Somewhat safe	Somewhat safe	Very safe	Somewhat safe	Somewhat safe	Neither safe nor unsafe	Somewhat safe	Neither safe nor unsafe
5. Describe the importance of diet in maintaining health in all ages, life-stages, sex and ethnic groups.	Very safe	Somewhat safe	Somewhat safe	Somewhat safe	Very safe	Somewhat safe	Somewhat safe	Very safe	Neither safe nor unsafe	Neither safe nor unsafe	Very safe	Somewhat safe	Somewhat safe	Very safe	Somewhat safe
6. Undertake a basic assessment of nutritional and hydration status to identify undernutrition, its consequences and treatment/ management.	Neither safe nor unsafe	Very unsafe	Neither safe nor unsafe	Neither safe nor unsafe	Somewhat unsafe	Somewhat safe	Very unsafe	Somewhat safe	Somewhat unsafe	Very safe	Somewhat safe	Somewhat safe	Somewhat safe	Neither safe nor unsafe	Somewhat unsafe
7. Identify individuals who are living with excess weight at all ages and the related health risk consequences and be aware of the treatment/management options, appreciating micronutrient deficien...	Very safe	Neither safe nor unsafe	Somewhat safe	Somewhat safe	Somewhat safe	Somewhat safe	Somewhat safe	Somewhat safe	Somewhat safe	Neither safe nor unsafe	Very safe	Somewhat safe	Somewhat unsafe	Somewhat safe	Somewhat unsafe
8. Have an understanding of, and know where to reference, the approximate average energy, macronutrient, water, micronutrient requirements and the more common deficiencies in the UK population	Neither safe nor unsafe	Very unsafe	Neither safe nor unsafe	Neither safe nor unsafe	Very unsafe	Neither safe nor unsafe	Neither safe nor unsafe	Neither safe nor unsafe	Neither safe nor unsafe	Very safe	Neither safe nor unsafe	Somewhat safe	Somewhat unsafe	Somewhat unsafe	Somewhat unsafe
9. Describe the official UK population dietary recommendations and be able to promote a "healthy balanced diet".	Very safe	Neither safe nor unsafe	Somewhat safe	Somewhat safe	Very safe	Somewhat safe	Somewhat safe	Somewhat safe	Very safe	Neither safe nor unsafe	Very safe	Somewhat safe	Somewhat safe	Very unsafe	Neither safe nor unsafe
10. Describe the indications for patients needing clinically assisted nutrition and hydration support (oral, enteral and parenteral); know the principles of administration and the potential compli...	Somewhat safe	Neither safe nor unsafe	Somewhat safe	Somewhat safe	Somewhat unsafe	Somewhat safe	Neither safe nor unsafe	Somewhat safe	Neither safe nor unsafe	Very safe	Somewhat safe	Somewhat safe	Somewhat unsafe	Somewhat safe	Neither safe nor unsafe
11. Understand when it is appropriate to refer for specialist nutrition/dietetic support and/or to specialist nutrition/weight management services.	Very safe	Very unsafe	Neither safe nor unsafe	Somewhat safe	Somewhat unsafe	Somewhat safe	Neither safe nor unsafe	Neither safe nor unsafe	Somewhat unsafe	Somewhat safe	Somewhat safe	Somewhat safe	Somewhat unsafe	Somewhat safe	Somewhat unsafe
How many hours do you think you have spent on nutrition education during the whole MBBS? Throughout the course, you will have received lectures on biochemistry e.g. carbohydrate metabolism: the di...	16-20 hours	5 hours or less	5 hours or less	6-10 hours	6-10 hours	6-10 hours	6-10 hours	16-20 hours	11-15 hours	5 hours or less	11-15 hours	5 hours or less	6-10 hours	6-10 hours	6-10 hours
What do you think is the ideal time to spend on nutrition education during the whole MBBS?	26+ hours	11-15 hours	16-20 hours	16-20 hours	6-10 hours	11-15 hours	16-20 hours	16-20 hours	21-25 hours	11-15 hours	16-20 hours	5 hours or less	11-15 hours	26+ hours	26+ hours
Thinking back over the nutrition education you received as an MBBS student, please highlight key experiences you recall. These may have been PBL scenarios, small group activities, online modules, ...	Online modules, lectures content, a lot was covered in metabolism component of the course	Year 3 online quiz module on qmplus	1 lectures year 3	Lots of biochemistry in first and second year. Some information on public health	Y3 public health lectures, diabetes lectures, diabetes focused PBLs, metabolism lectures in early years	PBLs, lectures on screening tools and deficiencies, online modules for deficiencies	During the Year 3 Public Health module, where we had lectures focusing on diet-related disease prevention [illegible]	Lectures in year 1 went through macro metabolism<br><br>Lectures mainly talked about the [illegible]	Y3 public health lectures Some PBL scenarios GP placement Y3 Endocrine Y3 placement [illegible]	During public health week in year 3, there was multiple lectures in smoking and obesity. These provided [illegible]	One lecture	pbl on diabetes and obesity cholesterol lecture	Lectures on feeding routes	Obesity in a very public health way (market drivers of obesity etc). Then biochemical level (insulin, glucose, [illegible]	These are the key experiences I recall: - Vitamin deficiency teaching in years 1&2, including PBLs on certain [illegible]

Questions \ Response ID	1	2	3	4	5	6	7	8
What further nutrition content would you suggest be included in the MBBS at QMUL? Please suggest specific topics; e.g., dietary advice, illnesses related to malnutrition, socio-economic insights t...	More insight into how to approach patients with eating disorders and how to best support them, more insight into healthy and actually applicable tips to give to patients, insight in where to refer for different dietary issues s	Understandin g healthy food options better. Being able to give more examples of foods that can be made for families of different ethnic backgrounds (e.g. Jewish, Islamic, Hindu, Persian. Etc). Also more ward-based and clinically based content about weight and management of weight. NOT E-LEARNING. This does not promote knowledge and curiosity, and does not promote using the knowledge in clinical practice.	Do more dietary workshops or lecture days	Dietary advice, assessment of nutrition and for later years referral	Dietary guidance taught is very outdated. Listening to podcasts by doctors and reading books on nutrition has taught me content not even discussed on the MBBS. EG ultra processed food<br><br>In addition to this, I never felt equipped to discuss simple steps patients can take to improve their diet.<br><br>"You need to improve your diet" - whilst factually correct- is not a particularly helpful statement for	learn more about obesity, factors that lead to it, how to advice patients, insight to different diets to recommend to patients, websites	Illnesses due to malnutrition, dietary history assessment	More diet comm stations are needed to try convince patients, more teaching about types of feeding needed
How far would you agree that nutrition education is important in medical school training? (1-10)	10	9	8	10	10	10	8	8
Overall, how well prepared do you feel to identify, advise, and refer patients for nutrition-related issues and illnesses? (0=Not at all prepared - 10 Extremely well prepared).	8	5	4	6	2	4	5	7
GMC Outcomes for graduates: Safeguarding vulnerable patients 7. Newly qualified doctors must be able to recognise and identify factors that suggest patient vulnerability and take action in response. Are you able to recognise where addiction (to drugs, alcohol, smoking or other substances), poor nutrition, self neglect, environmental exposure, or financial or social deprivation are [is} contributing to ill health. And take action by seeking advice from colleagues and making appropriate referrals?Statement 1	Most of the time	Yes, absolutely	Probably sufficiently	Most of the time	Probably sufficiently	Probably sufficiently	Probably sufficiently	Yes, absolutely
GMC Outcomes for graduates: Applying biomedical scientific principles. 22. Newly qualified doctors must be able to apply biomedical scientific principles, methods and knowledge to medical practice and integrate these into patient care. This must include principles and knowledge relating to nutrition. Based on your MBBS nutrition education how safe and confident would you feel to fulfil the criteria listed below:								
A. Explain how normal human structure and function and physiological processes applies, including at the extremes of age, in children and young people and during pregnancy and childbirth.	Somewhat safe	Very safe	Somewhat safe	Very safe	Somewhat unsafe	Somewhat safe	Neither safe nor unsafe	Somewhat safe
B. Explain the relevant scientific processes underlying common and important disease processes.	Somewhat safe	Very safe	Somewhat safe	Very safe	Very unsafe	Somewhat safe	Neither safe nor unsafe	Somewhat safe
C. Justify, through an explanation of the underlying fundamental principles and clinical reasoning, the selection of appropriate investigations for common clinical conditions and diseases.	Somewhat safe	Very safe	Somewhat safe	Somewhat safe	Very safe	Somewhat safe	Neither safe nor unsafe	Somewhat safe
D. Select appropriate forms of management for common diseases, and ways of preventing common diseases, and explain their modes of action and their risks from first principles.	Somewhat safe	Somewhat safe	Somewhat safe	Somewhat safe	Very safe	Somewhat safe	Somewhat safe	Very safe

Questions \ Response ID	9	10	11	12	13	14	15	16
What further nutrition content would you suggest be included in the MBBS at QMUL? Please suggest specific topics; e.g., dietary advice, illnesses related to malnutrition, socio-economic insights t...	It would be useful to have a quick screening strategy so whenever we clerk a patient we can consider a couple of basic questions that may indicate a need for a more thorough assessment. It might be nice to learn more about the options used by dieticians as we doctors may be involved in prescribing them, especially in the community.	Dietary assessment and plans How to give accessible advice re nutrition changes	dietary advice	Specific malnutrition in different ethnic groups Dietary advice holistically for different ethnic groups/ socioeconomi c classes Advice regarding consumption of seed oils compared with healthier fats eg butter. Advice re company marketing and mislabelling of food eg olive oils in supermarkets and the true proportion of olive oil in them. Advice/leaflet s re “healthier” options that are not actually healthy eg	Personal and Patient related diet advice. What makes up a healthy diet, how to lose weight and how to gain weight. Use practical workshops and offer the opportunity to do it with actual patients on the wards	cooking skills, dietary history assessmenT	info on dietary advice. especially how it can help in certain conditions such as pcos	I think it is covered pretty well, in terms of teaching opportunities. Nutrition based teaching may be the ones people are likely to switch off on/not attend, just because they feel it is not examined on/not important. If you had some more questions on it then people would be forced to revise. You could easily get people to fill out a must score and answer questions on nutrition in an osce.<br><br>People often
How far would you agree that nutrition education is important in medical school training? (1-10)	10	7	10	10	10	7	10	10
Overall, how well prepared do you feel to identify, advise, and refer patients for nutrition-related issues and illnesses? (0=Not at all prepared - 10 Extremely well prepared).	6	6	4	7	0	7	7	7
GMC Outcomes for graduates: Safeguarding vulnerable patients 7. Newly qualified doctors must be able to recognise and identify factors that suggest patient vulnerability and take action in response. Are you able to recognise where addiction (to drugs, alcohol, smoking or other substances), poor nutrition, self neglect, environmental exposure, or financial or social deprivation are [is} contributing to ill health. And take action by seeking advice from colleagues and making appropriate referrals?Statement 1	Most of the time	Most of the time	Little of the time	Probably sufficiently	Most of the time	Probably sufficiently	Most of the time	Most of the time
GMC Outcomes for graduates: Applying biomedical scientific principles. 22. Newly qualified doctors must be able to apply biomedical scientific principles, methods and knowledge to medical practice and integrate these into patient care. This must include principles and knowledge relating to nutrition. Based on your MBBS nutrition education how safe and confident would you feel to fulfil the criteria listed below:								
A. Explain how normal human structure and function and physiological processes applies, including at the extremes of age, in children and young people and during pregnancy and childbirth.	Somewhat safe	Neither safe nor unsafe	Somewhat unsafe	Somewhat safe	Somewhat safe	Somewhat safe	Somewhat safe	Somewhat safe
B. Explain the relevant scientific processes underlying common and important disease processes.	Very safe	Somewhat safe	Very unsafe	Somewhat safe	Somewhat safe	Somewhat safe	Somewhat safe	Somewhat safe
C. Justify, through an explanation of the underlying fundamental principles and clinical reasoning, the selection of appropriate investigations for common clinical conditions and diseases.	Very safe	Somewhat safe	Somewhat unsafe	Somewhat safe	Neither safe nor unsafe	Somewhat safe	Somewhat safe	Somewhat safe
D. Select appropriate forms of management for common diseases, and ways of preventing common diseases, and explain their modes of action and their risks from first principles.	Somewhat safe	Somewhat safe	Somewhat unsafe	Somewhat safe	Neither safe nor unsafe	Somewhat safe	Somewhat safe	Somewhat safe

Questions \ Response ID	17	18	19	20	21	22	23	24
What further nutrition content would you suggest be included in the MBBS at QMUL? Please suggest specific topics; e.g., dietary advice, illnesses related to malnutrition, socio-economic insights t...	Workshops about electrolytes replacement, about different styles of delivering nutrition e.g. parenteral, enteral and monitoring	Dietary advice managing malnutrition in hospital. Nutrition in primary care advice and practi e conversations with patients	Ward based tutorials on dietary needs and malnutrition	Practical dietery advice that can be given to patients. How it can be personalised to individuals with specific needs and preferences. Workshop style sessions. SSCs in nutrition. Debunking trending nutrition advice	Assessing nutrition status, lectures, workshops	Dietary counselling (not sure if this is a part of 4/5 year), catering management to dietary preferences<br><br>E-learning, ward based, online material like videos and modules, workshops	I would suggest including more clinically relevant and practical content. Specific topics could include: how to give tailored dietary advice for common conditions such as diabetes, obesity, cardiovascula r disease, and gastrointestin al disorders; the identification and management of illnesses related to malnutrition, including both undernutrition and overnutrition; and the influence of	Combating nutritional misinformatio n<br><br>How Gym Supplements affect the body<br><br>Combating food deserts<br><br>Diets that are protective from diverticulitis polyps and colon cancer.<br><br>More information about sweeteners<br><br>Way More information about postoperative nutrition
How far would you agree that nutrition education is important in medical school training? (1-10)	10	8	9	10	9	8	10	6
Overall, how well prepared do you feel to identify, advise, and refer patients for nutrition-related issues and illnesses? (0=Not at all prepared - 10 Extremely well prepared).	5	3	3	3	6	5	4	5
GMC Outcomes for graduates: Safeguarding vulnerable patients 7. Newly qualified doctors must be able to recognise and identify factors that suggest patient vulnerability and take action in response. Are you able to recognise where addiction (to drugs, alcohol, smoking or other substances), poor nutrition, self neglect, environmental exposure, or financial or social deprivation are [is} contributing to ill health. And take action by seeking advice from colleagues and making appropriate referrals?Statement 1	Yes, absolutely	Most of the time	Probably sufficiently	Probably sufficiently	Probably sufficiently	Probably sufficiently	Little of the time	Probably sufficiently
GMC Outcomes for graduates: Applying biomedical scientific principles. 22. Newly qualified doctors must be able to apply biomedical scientific principles, methods and knowledge to medical practice and integrate these into patient care. This must include principles and knowledge relating to nutrition. Based on your MBBS nutrition education how safe and confident would you feel to fulfil the criteria listed below:								
A. Explain how normal human structure and function and physiological processes applies, including at the extremes of age, in children and young people and during pregnancy and childbirth.	Very safe	Neither safe nor unsafe	Somewhat unsafe	Neither safe nor unsafe	Somewhat safe	Somewhat safe	Neither safe nor unsafe	Very safe
B. Explain the relevant scientific processes underlying common and important disease processes.	Very safe	Somewhat safe	Somewhat safe	Somewhat safe	Somewhat safe	Somewhat safe	Somewhat unsafe	Very safe
C. Justify, through an explanation of the underlying fundamental principles and clinical reasoning, the selection of appropriate investigations for common clinical conditions and diseases.	Very safe	Somewhat safe	Somewhat safe	Somewhat safe	Somewhat safe	Somewhat safe	Somewhat unsafe	Somewhat safe
D. Select appropriate forms of management for common diseases, and ways of preventing common diseases, and explain their modes of action and their risks from first principles.	Very safe	Very safe	Somewhat safe	Somewhat safe	Somewhat safe	Somewhat safe	Neither safe nor unsafe	Very safe

Questions \ Response ID	25	26	27	28	29	30	31
What further nutrition content would you suggest be included in the MBBS at QMUL? Please suggest specific topics; e.g., dietary advice, illnesses related to malnutrition, socio-economic insights t...	Nutritional assessments More focus on underweight assessments Illnesses related to malnutrition Relate to socio-economics	I think more logic advice on how to construct a diet for a specific patient type would be incredibly useful.	N/a	workshops?	Socio economic insights into dietary preference	Clear guidance on UK recommendat ion re diet, excercise. Also how to create a shared treatment plan than is stepped to introduce/re move foods in a way that patients are likely to follow.	I believe we should have more lectures on nutrition in medical school. Even though we have had a couple throughout medical school, it is nowhere near enough to grasp the importance of this topic in health. This should include a PBL/TBL where it gets more interactive and students need to do more research in their own time.<br><br>Further, there is a big issue surrounding talking about weight to
How far would you agree that nutrition education is important in medical school training? (1-10)	10	7	9	4	9	10	10
Overall, how well prepared do you feel to identify, advise, and refer patients for nutrition-related issues and illnesses? (0=Not at all prepared - 10 Extremely well prepared).	4	5	9	5	7	1	3
GMC Outcomes for graduates: Safeguarding vulnerable patients 7. Newly qualified doctors must be able to recognise and identify factors that suggest patient vulnerability and take action in response. Are you able to recognise where addiction (to drugs, alcohol, smoking or other substances), poor nutrition, self neglect, environmental exposure, or financial or social deprivation are [is} contributing to ill health. And take action by seeking advice from colleagues and making appropriate referrals?Statement 1	Most of the time	Yes, absolutely	Probably sufficiently	Most of the time	Most of the time	Most of the time	Little of the time
GMC Outcomes for graduates: Applying biomedical scientific principles. 22. Newly qualified doctors must be able to apply biomedical scientific principles, methods and knowledge to medical practice and integrate these into patient care. This must include principles and knowledge relating to nutrition. Based on your MBBS nutrition education how safe and confident would you feel to fulfil the criteria listed below:							
A. Explain how normal human structure and function and physiological processes applies, including at the extremes of age, in children and young people and during pregnancy and childbirth.	Very safe	Somewhat safe	Somewhat safe	Somewhat safe	Neither safe nor unsafe	Somewhat safe	Neither safe nor unsafe
B. Explain the relevant scientific processes underlying common and important disease processes.	Very safe	Neither safe nor unsafe	Somewhat safe	Somewhat safe	Somewhat safe	Very safe	Somewhat unsafe
C. Justify, through an explanation of the underlying fundamental principles and clinical reasoning, the selection of appropriate investigations for common clinical conditions and diseases.	Somewhat safe	Very safe	Somewhat safe	Somewhat safe	Somewhat safe	Very safe	Neither safe nor unsafe
D. Select appropriate forms of management for common diseases, and ways of preventing common diseases, and explain their modes of action and their risks from first principles.	Somewhat safe	Somewhat unsafe	Somewhat safe	Somewhat safe	Somewhat safe	Somewhat safe	Somewhat unsafe

Questions \ Response ID	1	2	3	4	5	6	7	8	9	10	11	12	13	14	15	16
E. Describe medications and medication actions: therapeutics and pharmacokinetics; medication side effects and interactions, including for multiple treatments, long term physical and mental condit...	Somewhat safe	Somewhat safe	Somewhat safe	Very safe	Very safe	Somewhat safe	Somewhat safe	Very safe	Somewhat safe	Somewhat safe	Somewhat safe	Somewhat safe	Neither safe nor unsafe	Somewhat safe	Somewhat safe	Neither safe nor unsafe
F. Analyse clinical phenomena and conduct appropriate critical appraisal and analysis of clinical data, and explain clinical reasoning in action and how they formulate a differential diagnosis and...	Somewhat safe	Somewhat safe	Somewhat safe	Somewhat safe	Very safe	Neither safe nor unsafe	Neither safe nor unsafe	Somewhat safe	Somewhat safe	Somewhat safe	Neither safe nor unsafe	Somewhat safe	Neither safe nor unsafe	Somewhat safe	Somewhat safe	Somewhat safe
GMC Outcomes for graduates: Health promotion and illness prevention. 25. Newly qualified doctors must be able to apply the principles, methods and knowledge of population health and the improvement of health and sustainable healthcare to medical practice. Based on your MBBS nutrition education how safe and confident would you feel to fulfil the criteria listed below as relates to nutrition and health:																
A. Explain the concept of wellness or wellbeing as well as illness, and be able to help and empower people to achieve the best health possible, including promoting lifestyle changes such as smokin...	Somewhat safe	Very safe	Somewhat safe	Somewhat safe	Very unsafe	Somewhat safe	Somewhat safe	Very safe	Somewhat safe	Somewhat safe	Neither safe nor unsafe	Somewhat safe	Very unsafe	Neither safe nor unsafe	Somewhat safe	Very safe
B. Describe the health of a population using basic epidemiological techniques and measurements.	Neither safe nor unsafe	Somewhat safe	Neither safe nor unsafe	Somewhat safe	Very unsafe	Neither safe nor unsafe	Somewhat safe	Somewhat safe	Very safe	Neither safe nor unsafe	Somewhat unsafe	Somewhat safe	Very unsafe	Somewhat safe	Somewhat safe	Somewhat safe
C. Evaluate the environmental, social, behavioural and cultural factors which infuence health and disease in different populations.	Somewhat safe	Very safe	Somewhat safe	Somewhat safe	Very unsafe	Somewhat safe	Somewhat safe	Somewhat safe	Very safe	Neither safe nor unsafe	Somewhat safe	Somewhat safe	Very unsafe	Neither safe nor unsafe	Somewhat safe	Somewhat safe
D. Assess, by taking a history, the environmental, social, psychological, behavioural and cultural factors influencing a patient's presentation, and identify options to address these, including advocacy for those who are disempowered.	Somewhat safe	Very safe	Very safe	Somewhat safe	Very unsafe	Somewhat safe	Somewhat safe	Very safe	Very safe	Somewhat safe	Somewhat safe	Somewhat safe	Very safe	Somewhat safe	Somewhat safe	Neither safe nor unsafe
E. Discuss the role and impact of nutrition to the health of individual patients and societies	Very safe	Neither safe nor unsafe	Neither safe nor unsafe	Somewhat safe	Very unsafe	Somewhat unsafe	Somewhat safe	Very safe	Very safe	Somewhat safe	Somewhat unsafe	Somewhat unsafe	Neither safe nor unsafe	Somewhat safe	Somewhat safe	Very safe
F. Evaluate the determinants of health and disease and variations in healthcare delivery and medical practice from a global perspective and explain the impact that global changes may have on local...	Somewhat safe	Somewhat safe	Neither safe nor unsafe	Neither safe nor unsafe	Very unsafe	Neither safe nor unsafe	Neither safe nor unsafe	Very safe	Somewhat safe	Neither safe nor unsafe	Somewhat unsafe	Somewhat safe	Somewhat unsafe	Somewhat safe	Somewhat safe	Somewhat safe
How far would you agree that poor diet is the number one killer of people in the UK? (0 Not agree at all - 10 Strongly agree).	7	7	8	7	9	5	5	9	7	7	6	10	9	6	6	7
This 5 minute video from the US presents a particular view on nutrition training for doctors, based on selected academic papers. Please watch it and make any comments on ideas presented in the text box below. https://youtu.be/ZRX2x9FnUg0	It is very sad that there is poor confidence in the ability of doctors to help patients with dietary problems. Nonetheless this public perception is actually	I agree with the core message, however the video makes the error of showing cherry picked data and does not delve into the quality of the	It is very interesting fact that diet is a leading killer in America yet it is not taken seriously, it is an issue that should be addressed worldwide	Very valid points. I believe that talking about nutrition and diet can be difficult and these days often more than smoking as it can be sensitive	Agreed	I was shook to learn that American diet is the no 1 cause of death	Even if doctors don't have the knowledge themselves they should be shown how to refer appropriately	None to think of	A comparison was made with stopping smoking. While there are loads of accessible smoking services available and tools/medic	-	Agree!!	Diet can reverse CAD is surprising and i wonder how biochemically. We were always taught it was irreversible I wonder how to specialise in lifestyle	Excellent video highliting great resources to learn more on such an important topic. I wasn't sure that poor diet was the number one cause of	Doctors typically receive under 20 hours of nutrition training—mostly biochemistry—while clinical nutrition time is often less than	i agree that we do not get enough training on nutrition.	I think we all have a poor understanding of nutrition across the globe, people and doctors alike. The video is probably too optimistic
Having watched the video, how far do you agree with the message that there is insufficient emphasis on nutrition in medical training generally?	8	8	10	10	10	10	6	8	10	8	10	10	10	6	10	8
These organisations promote the teaching of nutrition for doctors. Please tick any have you heard of.<br>1. Association for Nutrition (AfN) Undergraduate Curriculum in Nutrition for Medical Doctors<br>2. Nutritank<br>3. NNEdPro Global Institute for Food, Nutrition and Health<br>4. Culinary Medicine UK<br>5. Education and Research In Medical Nutrition Network (ERIMNN)<br>6. The Nutrition Implementation Coalition<br>7. None of the above.	None of the above;	Association for Nutrition (AfN) Undergraduate Curriculum in Nutrition for Medical Doctors ;	None of the above;	None of the above;	None of the above;	None of the above;	None of the above;	Nutritank;	None of the above;	Nutritank;	None of the above;	Nutritank;	None of the above;	The Nutrition Implementation Coalition;Nutritank;Culinary Medicine UK;	None of the above;	Association for Nutrition (AfN) Undergraduate Curriculum in Nutrition for Medical Doctors ;

Questions \ Response ID	17	18	19	20	21	22	23	24	25	26	27	28	29	30	31
E. Describe medications and medication actions: therapeutics and pharmacokinetics; medication side effects and interactions, including for multiple treatments, long term physical and mental condit...	Very safe	Very safe	Somewhat unsafe	Somewhat safe	Somewhat unsafe	Somewhat safe	Very unsafe	Somewhat safe	Somewhat safe	Very safe	Somewhat safe	Somewhat safe	Neither safe nor unsafe	Somewhat safe	Somewhat unsafe
F. Analyse clinical phenomena and conduct appropriate critical appraisal and analysis of clinical data, and explain clinical reasoning in action and how they formulate a differential diagnosis and...	Very safe	Very safe	Neither safe nor unsafe	Somewhat safe	Somewhat unsafe	Somewhat safe	Somewhat unsafe	Somewhat safe	Somewhat safe	Neither safe nor unsafe	Somewhat safe	Somewhat safe	Neither safe nor unsafe	Somewhat safe	Neither safe nor unsafe
GMC Outcomes for graduates: Health promotion and illness prevention. 25. Newly qualified doctors must be able to apply the principles, methods and knowledge of population health and the improvement of health and sustainable healthcare to medical practice. Based on your MBBS nutrition education how safe and confident would you feel to fulfil the criteria listed below as relates to nutrition and health:															
A. Explain the concept of wellness or wellbeing as well as illness, and be able to help and empower people to achieve the best health possible, including promoting lifestyle changes such as smokin...	Very safe	Somewhat safe	Somewhat safe	Somewhat safe	Somewhat safe	Somewhat safe	Somewhat safe	Very safe	Somewhat safe	Very safe	Somewhat safe	Somewhat safe	Somewhat safe	Somewhat unsafe	Somewhat unsafe
B. Describe the health of a population using basic epidemiological techniques and measurements.	Very safe	Somewhat unsafe	Somewhat safe	Somewhat safe	Somewhat unsafe	Somewhat safe	Neither safe nor unsafe	Neither safe nor unsafe	Somewhat unsafe	Neither safe nor unsafe	Somewhat safe	Somewhat safe	Neither safe nor unsafe	Somewhat safe	Neither safe nor unsafe
C. Evaluate the environmental, social, behavioural and cultural factors which infuence health and disease in different populations.	Very safe	Somewhat safe	Somewhat safe	Somewhat safe	Somewhat safe	Somewhat safe	Somewhat safe	Very safe	Very safe	Neither safe nor unsafe	Somewhat safe	Somewhat safe	Neither safe nor unsafe	Very safe	Neither safe nor unsafe
D. Assess, by taking a history, the environmental, social, psychological, behavioural and cultural factors influencing a patient's presentation, and identify options to address these, including advocacy for those who are disempowered.	Very safe	Very safe	Somewhat safe	Somewhat safe	Very safe	Somewhat safe	Somewhat unsafe	Somewhat safe	Somewhat safe	Very safe	Somewhat safe	Somewhat safe	Somewhat safe	Somewhat safe	Neither safe nor unsafe
E. Discuss the role and impact of nutrition to the health of individual patients and societies	Very safe	Somewhat safe	Somewhat safe	Neither safe nor unsafe	Very safe	Somewhat safe	Neither safe nor unsafe	Very safe	Somewhat safe	Neither safe nor unsafe	Somewhat safe	Somewhat safe	Somewhat safe	Somewhat safe	Somewhat unsafe
F. Evaluate the determinants of health and disease and variations in healthcare delivery and medical practice from a global perspective and explain the impact that global changes may have on local...	Very safe	Somewhat unsafe	Somewhat safe	Neither safe nor unsafe	Very safe	Somewhat safe	Somewhat safe	Very safe	Somewhat safe	Very safe	Somewhat safe	Somewhat safe	Somewhat safe	Somewhat safe	Somewhat unsafe
How far would you agree that poor diet is the number one killer of people in the UK? (0 Not agree at all - 10 Strongly agree).	7	7	9	8	7	5	8	8	3	8	8	3	8	6	10
This 5 minute video from the US presents a particular view on nutrition training for doctors, based on selected academic papers. Please watch it and make any comments on ideas presented in the text box below. https://youtu.be/ZRX2x9FnUg0	I agree with the video	Diet related diseases are highly prevalent however patients aren't being advised about nutritions effect on disease. Too much information	Diet has a huge contribution to our health	Much of the diseases we face are linked to nutrition. However, doctors aren't not taught or trained to address nutrition in the same way as	Poor diet is a huge issue globally and especially in the west. Yet doctors don't receive enough nutrition training.	Definitely agree with the ideas, nutrition counselling would form a huge part of preventative medicine and with the current focus it's important	- our diet determines our overall health (we are what we eat)<br>- 60% Americans - suffer from diet related issues due to lack of education	Nutrition is dependent on the patient to have an active approach to their diet I think doctors may get used to the idea that when these measures	N/a	Most medical students receive less than 20 hours of nutrition education across their entire training, often focused on nutrient	That despite nutritional issues being a major cause of a lot of disease, there's limited teaching in medical school, considering things like	realise we don't know a lot and that's worrying as nutrition is health	Blames poor nutrition for disease in most cases - however it is not the only important factor to consider	I absolutely agree that there is a mismatch between the perceived level of knowledge about lifestyle of diet by doctors, and the confidence	I find it really interesting how salt plays such a massive role in cardiovascular disease - and from shadowing cardiologists we hear advice to
Having watched the video, how far do you agree with the message that there is insufficient emphasis on nutrition in medical training generally?	10	8	4	10	8	8	10	8	7	7	8	7	6	10	10
These organisations promote the teaching of nutrition for doctors. Please tick any have you heard of.<br>1. Association for Nutrition (AfN) Undergraduate Curriculum in Nutrition for Medical Doctors<br>2. Nutritank<br>3. NNEdPro Global Institute for Food, Nutrition and Health<br>4. Culinary Medicine UK<br>5. Education and Research In Medical Nutrition Network (ERIMNN)<br>6. The Nutrition Implementation Coalition<br>7. None of the above.	Nutritank;	None of the above;	None of the above;	None of the above;	None of the above;	Nutritank;	None of the above;	Association for Nutrition (AfN) Undergraduate Curriculum in Nutrition for Medical Doctors ;	None of the above;	None of the above;	Nutritank;Association for Nutrition (AfN) Undergraduate Curriculum in Nutrition for Medical Doctors ;	None of the above;	None of the above;	None of the above;	None of the above;
