# Supplementary material for: Persistent gaps in nutrition education in UK medical schools: a triangulated review of curricula, student perception and the evidence base
Source: BMJ Nutr Prev Health. 2026 Apr 20;9(1):e001479. doi: 10.1136/bmjnph-2025-001479 (PMC13425111; doi:10.1136/bmjnph-2025-001479)
Supplement: online supplemental file 6 [file bmjnph-9-1-s006.pdf]

Study Characteristics						Aims		Population		Approach & Findings					
**DOI Link**	**Authors**	**Title**	**Article Type**	**Year of Publication**	**Location**	**Study Aims**	**Specific Focus**	**No of participants**	**Population description**	**Approach**	**Approach - detailed**	**Key findings**	**Key recommendations**	**Limitations**	
http://dx.doi.org/10.1017/S1368980018000800	Broad, Jonathan; Wallace, Megan	Nutrition and public health in medical education in the UK: reflections and next steps	Commentary	2018	UK	To comment on the gap in UK medical students' understanding of nutrition and public health, and suggest ways to improve it.	Literature review of nutrition education in medical schools and evaluations of a 6-week elective in public health nutrition for medical students	15 final year medical students were enrolled in the elective. 60 school pupils participated in the public health activity.	Medical school students. Local school students	Commentary. Review.	To outline current best practice in undergraduate education about nutrition, where medical schools fall behind, and discuss a nutrition curriculum in our medical school that incorporates active health promotion outreach into a local school.<br><br>**The curriculum pilot:** The 6-week elective course utilised a variety of approaches, including traditional small group lectures, workshops with student activities driving the learning, and practicals where students designed a public health intervention. Doctors, dietitians and clinical psychologists led the teaching. Subsequently students led a healthy eating workshop in a local school.	**Review:** A recent European-wide survey found that UK medical schools teach an average of 22 h of nutrition throughout the medical curriculum, slightly lower than the European average but higher than the US average<br><br>**Pilot Curriculum:** Following the course students felt more confident about nutrition (paired improvement =0·84, P=0·049) and had an improvement on a multiple-choice question about nutrition from 4·5 to 9 out of 10 (Wilcoxon signed rank W <0·001). In a focus group, students felt that the curriculum was relevant to their future work and felt more confident about managing in practice.	Enabling students to understand the individual and social determinants of food and diet will enable our cohort to better understand, prevent and treat the health consequences of malnutrition and obesity.<br><br>Expansion of current teaching in health professionals is necessary to meet the large burden of nutrition-related disease. This will support doctors to meet the General Medical Council's expectations and equip future health professionals with the skills to manage poor nutrition and promote healthy eating in the patient population.	None listed for review.<br><br>For the pilot curriculum, limitations listed were the length of the course (short 6 week elective) and the uptake (would like more students to complete this)	
https://doi.org/10.1136/bmjnph-2022-000513	Blythe, J., Eden, T., Macaninch, E., Martyn, K., Ray, S., Patel, N., & Fernandes, K	Case studies and realist review of nutrition education innovations within the UK medical undergraduate curricula	Article	2022	UK	To evaluate current curriculum innovations within UK medical training	To provide case studies of innovative nutrition education interventions in UK medical schools, describing how they were designed and delivered. To use a realist review approach to understand why, how, and in what contexts these interventions work (or don't), in order to draw out lessons for practice.	228 students (Case study 1 - working with Third sector);<br>240 students (Case Study 2 - e-learning)	Medical school students	Realist review, case studies	Presents 2 case studies.<br><br>**Case Study 1**<br>**Barts Medical School p**artnered with Bags of Taste, a community-based organisation that runs cooking classes for people in poverty, using a behaviour-change model. Year 3 medical students (n = 228) spent a half-day in small groups (max 12) with Bags of Taste in a community environment. Students observed cooking, participated, and discussed food poverty. After the session, students took home a bag of ingredients (same as the Bags of Taste participants) and were invited to cook the recipe at home. Students wrote reflections on what they learned, especially around	Across the case studies and the realist review, the key themes were clear.<br><br>a) Curriculum issues<br>The medical curriculum is already "packed," so adding new content is challenging. Opportunities to insert nutrition teaching may arise opportunistically (e.g., when there is free time in teaching schedules) or as part of structured curriculum review.<br><br>b) Legitimacy and Visibility<br>Nutrition historically has had low visibility in medical education; some stakeholders don't see it as "core." Appointing Nutrition Leads / Champions helps raise the profile: their presence gives legitimacy and institutional	The authors call for further research: particularly into interprofessional education models, scaling up innovations, and measuring long-term impacts (both on student behaviour and patient outcomes). There is a need for national / external accreditation of nutrition education modules to strengthen their legitimacy and ensure consistent quality. Student involvement is crucial: students can co-design, evaluate, and champion nutrition education in ways that faculty alone may not.	Case studies are limited to two settings. Evaluation lies heavily on student self-report, and is short term. No long term outcome data. Sustainability is a concern - the e-learning module was short term and one off.	
https://doi.org/10.1093/advances/nmy082	Blunt, Stavia; Kafatos, Anthony	Clinical Nutrition Education of Doctors and Medical Students: Solving the Catch 22	Commentary	2019	Europe	To highlight the gap in nutrition education among doctors and medical students. To identify barriers to effective nutrition training in medicine, and suggest possible educational approaches to overcome them	Commentary that explores the "Catch-22", which is that medical educators themselves lack adequate nutrition training, which prevents them from training the next generation. Also discusses the broader public health implications of malnutrition and the role doctors could play.	N/A	N/A	Narrative commentary.	The authors draw on existing literature from across Europe, their own experiences and illustrative examples of nutrition education programs. They discuss structural issues and propose solutions.	Barriers to nutrition education in medicine include: insufficient curricular time; historical bias toward disease and pharmacotherapy rather than prevention and diet; lack of confidence among physicians; unclear role definitions on medical teams; and public confusion about who is responsible for giving nutrition advice. There are very few qualified "nutrition teachers" in medicine; without them, it's difficult to train the next generation. Some successful educational approaches do exist, but they are limited, and their effectiveness is not yet fully evaluated.	A call for urgent scale-up of nutrition training at all levels: medical students, trainees, practicing physicians.<br>Develop and support proper nutrition "train-the-trainer" programs to build capacity among medical educators.<br>Incorporate internationally accessible nutrition education programs, to overcome the "Catch-22" of lacking qualified trainers.<br>Use illustrative successful programs as models and evaluate them rigorously to identify best practices.	Not empirical - no data collection, a commentary. Hihglights only selected examples, rather than systematic review.	
http://dx.doi.org/10.3390/nu13030957	Ganis, Laura; Christides, Tatiana	Are We Neglecting Nutrition in UK Medical Training? A Quantitative Analysis of Nutrition-Related Education in Postgraduate Medical Training Curriculums	Article	2021	UK	To evaluate current teaching on nutrition in postgraduate medical education.	To (1) Quantify nutrition-related learning objectives (NLOs) in UK postgraduate medical training curriculums and assess variation across specialties; (2) assess inclusion of nutrition-related modules; (3) assess the extent to which NLOs are knowledge-, skill-, or behaviour-based, and in which Good Medical Practice (GMP) Domain(s) they fall.	N/A	N/A	Review of curriculums and learning objectives	Between August and October 2020, one investigator independently reviewed the published curriculums of 43 UK postgraduate medical training programmes in the UK. Curriculums were searched for nutrition learning objective. These NLOs were compiled in a spreadsheet and tallied. Objectives were coded as skill or behaviour based. Where possible, they were also coded with a GMP domain. Spearman's Rank Correlation Coefficient (Rs) was used to assess the relationship between keywords and GMP Domains. For each curriculum, the proportion of NLOs identified by each keyword was ranked against the proportion of NLOs designated to each Domain; for example, in the Core	All 43 curriculums had at least one NLO identified, with a median of 15 NLOs per curriculum. Range of NLOs was 1 - 177. Surgical curriculums had a higher number of identified NLOs compared with medical curriculums. Across specialties, the most common keyword in identified NLOs was 'nutrition' (513 objectives), followed by 'obesity' (229), 'lifestyle' (166), and 'diet' (122). In 22 specialities (51%) at least one nutrition related module was identified, with a maximum of 13 modules in a single curriculum. Modules were identified in 100% of surgical curriculums (mean 2.7 modules per curriculum) compared with 34.4% of medical curriculums (mean 0.8 modules per curriculum). 84% of NLOs identified across	Action-oriented research should be used to address identified barriers which hinder doctors in recognising and effectively treating malnutrition, a major cause of mortality and morbidity in the UK. The findings in this study suggest the need for a standardised approach to nutrition education, which fundamentally integrates a generic set of nutrition-related objectives across all postgraduate medical curriculums, in line with GMC frameworks for excellence. This study provides evidence<br>of the need for renewed focus on communication skills and professional behaviours to ensure that all doctors feel equipped to help patients achieve or	(a) The quantitative focus of this study design. Firstly, the number of NLOs in a curriculum bears no reflection on actual nutrition-related practice within an identified medical field, and authors are therefore unable to comment on how variation in nutrition-related outcomes in curriculums translates into variation in practice. (b) Secondly, a full thematic analysis of all objectives was outside the scope of this study. (c) Finally, in postgraduate training there is an assumption that all curricular	
http://dx.doi.org/10.1136/flgastro-2022-102089	Jones, Glenys; Craigie, Angela M.; Zaremba, Suzanne M. M.; Jaffee, Ally; Mellor, Duane D.	Teaching medical students about nutrition: from basic principles to practical strategies	Commentary	2023	UK	To discuss three key factors for successful and achievable nutrition education in medical schools.	This paper discusses three key ways in which medical schools can support the implementation of nutrition into their teaching; incorporating nutrition within the core medical curriculum teaching, the use of subject specific experts to support and deliver nutrition training, and the inclusion of nutrition within formal assessment so as to reinforce and cement learnings into practical, applicable actions and advice.	N/A	N/A	Commentary.	Commentary with three recommendations for improving medical education on nutrition in the UK. Aligns with The Association for Nutrition UK Undergraduate Curriculum in Nutrition for Medical Doctors, which provides medical schools with guidance on what should be included in the training of all medical students	The undergraduate curriculum has appeared to lag behind the postgraduate specialty curricula of the colleges, with respect to clear structure and learning outcomes on nutrition. However, this perhaps reflects the broader nature of undergraduate education and an already very busy curriculum. Despite these challenges, there are three suggested factors to improve the curriculum:<br>(1) Incorporate within the core curriculum<br>(2) Use the knowledge and skills of subject specialists (e.g. registered nutritionists)<br>(3) Include nutrition within assessments and practical clinical examinations	The time is now here for medical schools to demonstrate their innovative and forward-thinking training, by integrating the AfN UK Undergraduate Curriculum in Nutrition for Medical Doctors19 into their teaching provision and preparing their graduates with the knowledge and skills needed to aid their practice and improve patient care.	N/A	
http://dx.doi.org/10.1017/S0007114522001635	Jones, Glenys; Macaninch, Elaine; Mellor, Duane D.; Spiro, Ayela; Martyn, Kathy; Butler, Thomas; Johnson, Alice; Moore, J. Bernadette	Putting nutrition education on the table: development of a curriculum to meet future doctors' needs	Article	2023	UK	To develop a new, modern undergraduate nutrition curriculum for medical doctors.	The AfN IPG brought together expertise from nutrition, dietetic and medical professionals, representing the National Health Service (NHS), royal colleges, medical schools and universities, government public health departments, learned societies, medical students, and nutrition educators. The curriculum was developed with the key objective of being implementable through integration with the current undergraduate training of	N/A	N/A	Curriculum development	The AfN formed an Interprofessional Working Group on Medical Education (AfN IPG). The group brought together expert professionals and organisations, as well as those who would play a key role in delivery of, or be influenced by, the updated curriculum. The working group represented Public Health England, NHS England, nutrition and dietetic professionals, medical royal colleges, medical schools, medical students, doctors and training providers. The nutrition curriculum was developed through collaborative and open discussion between group members over a number of meetings, with an agreement reached by consensus over the required detail and structure.	A consensus-led, multi-stakeholder process led by the AfN has developed a modern nutrition curriculum for undergraduate medical students in the UK. The AfN IPG recommends that medical schools deliver **thirteen core nutritional competencies** in order for future medical doctors to master eleven graduation fundamentals in nutrition by the point of their graduation.<br><br>A potential challenge raised by medical schools will be how to incorporate, what to some may first appear to be additional material, nutrition into an already incredibly dense curriculum. Clearly signposting to where nutritional science already exists in undergraduate medical foundational training	It is now imperative that nutrition fundamentals be embedded in core undergraduate training for medical doctors. There is a clear opportunity now for medical schools to distinguish themselves based on the integration of nutrition practice into holistic healthcare training to adequately prepare graduates with the knowledge and skills in nutrition care with the ultimate goal of improving patient care.	None listed.	

Study Characteristics						Aims		Population		Approach & Findings				
**DOI Link**	**Authors**	**Title**	**Article Type**	**Year of Publication**	**Location**	**Study Aims**	**Specific Focus**	**No of participants**	**Population description**	**Approach**	**Approach - detailed**	**Key findings**	**Key recommendations**	**Limitations**
http://dx.doi.org/10.1111/jhn.70031	Khiri N, Howells K	Nutritional Education in Medical Curricula and Clinical Practice: A Scoping Review on the Knowledge Deficit Amongst Medical Students and Doctors	Article	2025	Global	To synthesize the literature on nutrition education for medical students and doctors in English-speaking countries. To identify why there is a gap in nutrition knowledge and skills. To propose solutions to close that gap.	To review exisiting literature and interventions (e.g. curriculur changes) to better understand and evaluate existing innovations, gaps and next steps.	N/A	N/A	Scoping review following PRISMA-ScR guidelines.	Databases searched: PubMed, Web of Science, Embase, ERIC; plus grey literature (Google, Bing, Perplexity AI).<br>Time frame: articles from 2014 to 2024.<br>Thematic analysis: Braun & Clarke's six-step approach.<br>Coding software: Delve qualitative coding tool.<br>PICO tool used to help define the review question	**Identified four main reasons for the nutrition knowledge gap:**<br>1. Insufficient curriculum time for nutrition.<br>2. Perceptions and confidence: MSAD may feel nutrition is unimportant, or lack confidence to counsel.<br>3. Stigma and health habits: the sensitivity of diet, weight, and nutrition discussions; also personal health behaviors of doctors.<br>4. Challenges in clinical practice: limited time, lack of protocols, unclear referral pathways (e.g., dietitian).<br><br>**Proposed four solution themes:**<br>1. Curriculum innovations (integrate nutrition throughout training)<br>2. Standardization via competency standards	Introduce curricular innovations so nutrition education is longitudinal throughout medical training (undergrad, postgraduate).<br><br>Develop and implement national (or international) competency standards in nutrition for doctors.<br><br>Strengthen postgraduate training pathways in nutrition.<br><br>Encourage collaboration between dietitians and medical educators to teach nutrition.<br><br>Increase integration of nutrition into day-to-day clinical practice, not just in theoretical	Only includes English-speaking countries (limiting generalizability). Variability in quality and design of the included studies (since the 28 papers used different methods). The use of thematic analysis means findings are necessarily interpretive and may be influenced by coding decisions.
http://dx.doi.org/10.1136/bmjnph-2023-000807	Lepre B,Mansfield KJ,Ray S,Beck EJ	Establishing consensus on nutrition competencies for medicine: a Delphi study	Article	2024	Global	To establish a consensus on what nutrition competencies doctors (medical practitioners) should have. To develop a framework of competencies to guide nutrition education in medical training.	To gain input from experts (practitioners, educators, policymakers, service users) to define core nutrition competencies that are relevant for screening, assessment, coordination of care, and interprofessional work.	Experts panel: 52 experts in Round 1 (15.1% response rate), 42 in Round 2, 47 in Round 3. Service-users panel (patients / public): 27 in Round 1 (57.5% response), 19 Round 2, 16 Round 3.	The expert group included: medical professionals, dietitians, health professions education academics, policymakers from Australia, New Zealand, UK, Northern Ireland.	Three-round modified online Delphi survey	Participants rated proposed competencies, provided feedback, and refined until consensus.	Achieved consensus on 25 nutrition competencies for medicine. The service-user panel (public) added 7 additional skills/attributes considered important for receiving nutrition care. Competencies clustered around themes: team-based care, communication, professionalism, health promotion and disease prevention. Competencies reflect not just knowledge, but also the need to coordinate care and refer when appropriate.	The primary recommendation is to use these competencies as a benchmark to inform curriculum development in medical education. Also, incorporate training so that doctors can: perform nutrition screening; interpret assessments; coordinate with other professionals; support patient advice given by dietitians. Also, to embed the competencies into accreditation, education standards, and assessments to ensure they are adopted.	Response rate from experts was relatively low (15.1% in Round 1), which might limit representativeness. Delphi panel largely from a limited geographic region (Australia, NZ, UK, Northern Ireland), so competencies may not fully generalize globally. Some competencies may overlap with non-nutrition competencies (e.g., communication, teamwork), making integration into curricula complex. The process defines "what should be," but does not test how well training on these competencies
http://dx.doi.org/10.1136/bmjopen-2020-043066	Lepre B,Mansfield KJ,Ray S,Beck E	Nutrition competencies for medicine: an integrative review and critical synthesis	Article	2021	Global	To synthesize existing nutrition competency frameworks for medical education globally. To critically analyze and compare them, and propose a consolidated competency framework (Nutrition Competency Framework, NCF) for medicine.	The study was focused on published nutrition competency frameworks across different contexts, and identifying common themes across frameworks and gaps	N/A	N/A	Integrative Review	Searched multiple databases (CINAHL, Medline, Embase, Scopus, Web of Science, Global Health) through April 2020.<br>Hand-searching of references to find additional frameworks.<br>Data extraction into summary tables, then thematic analysis (identifying common themes).<br>Used theoretical models to interpret competence: Miller's pyramid (knows, knows how, shows how, does), Knowledge to Action Cycle, Dreyfus model of skill acquisition.	Identified 25 unique nutrition competencies relevant for medical education.<br>Competencies grouped into five major themes:<br><br>1. Clinical practice<br>2. Health promotion & disease prevention<br>3. Communication<br>4. Working as a team<br>5. Professional practice (professionalism)<br><br>Majority of the competencies are knowledge-based; fewer are skills-based, and even fewer are attitude/value-based.	Vertical integration of nutrition competencies into the medical curriculum: embed nutrition across all years, not just in isolated modules.<br><br>Increase opportunities for skill-based training (not just knowledge) — for example, clinical assessments, case-based learning, OSCEs (Objective Structured Clinical Examinations).<br><br>Use the 25 competencies as a benchmark / reference for curriculum developers, accreditation bodies, and educators.<br><br>Encourage ongoing development and validation of the nutrition competency	The search was not systematic, and may have missed relevant frameworks.<br>Extraction of data was done by a single author in some cases, increasing risk of bias.<br>The included literature is heavily skewed towards the USA and other high-income settings; less representation from low- or middle-income countries.<br>The proposed framework is conceptual; it has not been empirically validated across different medical schools.
http://dx.doi.org/10.1136/bmjnph-2021-000234	Lepre B,Mansfield KJ,Ray S,Beck EJ	Reference to nutrition in medical accreditation and curriculum guidance: a comparative analysis	Article	2021	Global	To assess how nutrition is represented in medical accreditation standards and curriculum guidance documents internationally. To identify whether nutrition requirements exist in formal regulatory frameworks, which could drive its inclusion in curricula.	To explore the presence (or absence) of explicit nutrition content in: accreditation standards, competency frameworks, curricula, assessments. To make a comparison across countries.	N/A	N/A	Non-systematic comparative analysis.	The authors conducted internet searches including Google Search, WHO Directory of Medical Schools, Foundation for Advancement of International Medical Education and Research (FAIMER) directory.<br>ncluded documents: accreditation standards, competency frameworks, curricula, assessment guidance.<br>Stratified findings by country / region	Only 44% of the accreditation / curriculum guidance documents reviewed included any nutrition content. Nutrition is inadequately represented in formal regulatory frameworks at many levels. Where nutrition is included, it is not always mandatory or enforced, limiting its integration into curricula.<br><br>The authors included 7 documents from the UK & Northern Ireland (accreditation and curricular guidance). Of those, only 3 contained any reference to nutrition (i.e., ~43%) in their standards or guidance. The paper notes that the General Medical Council (GMC)'s 2018 Outcomes for Graduates includes explicit nutrition-related outcomes. But, only a small number of assessment /	Advocate for embedding nutrition into accreditation standards, so that medical schools are required (not optional) to teach nutrition.<br>Regulatory bodies, governments, and educational institutions should work together to ensure nutrition is recognized as a core competency.<br>Use policy levers ("top-down" approaches) to incentivize integration of nutrition into curricula, rather than relying solely on individual institutions.	The primary limitation is that the review is non-systematic. Publicly available documents may not reflect internal or unpublished curricular practices; what's "on paper" may differ from actual teaching. Also, the analysis focuses on presence/absence rather than the quality or depth of nutrition content.
http://dx.doi.org/10.1136/bmjnph-2019-000049	Macaninch, Elaine; Buckner, Luke; Amin, Preya; Broadley, Iain; Crocombe, Dominic; Herath, Duleni; Jaffee, Ally; Carter, Harrison; Golubic, Rajna; Rajput-Ray, Minha; Martyn, Kathy; Ray, Sumantra	Time for nutrition in medical education	Article	2022	UK	To synthesise a selection of UK medical students' and doctors' views surrounding nutrition in medical education and practice.	This paper contributes to the landscaping of need within medical nutrition training through summarising the observations of survey data and a UK medical school curriculum review that analyse the beliefs of medical students and junior doctors on the standard of nutrition teaching in medical education.	853	Pooled from 5 heterogenous sources.	Survey study of perspectives	Information was gathered from surveys of medical students and doctors identified between 2015 and 2018 and an evaluation of nutrition teaching in a single UK medical school. Comparative analysis of the findings was undertaken to answer three questions: the perceived importance of nutrition in medical education and practice, adequacy of nutrition training, and confidence in current nutrition knowledge and skills.	Most agreed on the importance of nutrition in health (>90%) and in a doctor's role in nutritional care (>95%). However, there was less desire for more nutrition education in doctors (85%) and in medical students (68%). Most felt their nutrition training was inadequate, with >70% reporting less than 2 hours. There was a preference for face-to-facerather than online training. At one medical school, nutrition was included in only one module, but this increased to eight modules following an increased nutrition focus. When medical students were asked about confidence in their nutrition knowledge and on advising patients, there was an even split between agree and disagree (p=0.869 and p=0.167, respectively), yet few were	One such area that requires further exploration is how different professional groups, including doctors, perceive their role in providing nutritional care. This is important to identify professional responsibilities within nutritional care pathways, including where aspects are likely to be missed. While there is a clear need to improve nutritional care in both primary and secondary care settings, it is essential to provide focus on the development of nutrition education resources that support new students' and existing doctors' professional nutrition development.<br><br>There are less recommendations - this is	As a collection these surveys and evaluations have not been validated and all suffer from selection bias, as by nature those most likely to respond and complete the surveys will have an interest in this topic. In addition, participants are likely to assume that the survey was designed to increase nutrition content, and therefore they may tailor their answers to align in this fashion. Further to this the lack of standardisation between surveys and evaluation techniques means there is less comparability between reviews,
http://dx.doi.org/10.1111/jhn.12972	Patel P,Kassam S	Evaluating nutrition education interventions for medical students: A rapid review	Article	2022		To evaluate the efficacy and characteristics of nutrition education interventions aimed at undergraduate medical students.<br>To assess what methods are being used, how long interventions last, and how outcomes are measured.	Explored nutrition curriculum interventions within a set time frame (2015-2020) and evaluated the quality of the interventions against set educational criteria.	N/A	N/A	Rapid Review	Searched literature published between 2015 and 2020.<br>Outcomes evaluated using the modified Kirkpatrick hierarchy (levels of training evaluation).<br>Quality appraisal of studies using the MERSQI (Medical Education Research Study Quality Instrument).<br>Narrative synthesis of study characteristics and outcomes.	Wide heterogeneity in interventions (types, duration, content).<br>Interventions included cooking sessions, lectures, student-led programs.<br>Median duration was 11 hours, ranging from 90 minutes to 75 hours.<br>Modified Kirkpatrick scores (measuring impact) varied across interventions.<br>Median MERSQI score of 12.8/18, indicating moderate methodological quality.<br>None of the studies used national or standardized guidance (e.g., no unified competency framework) to design their interventions	Use interprofessional learning (IPL) in nutrition education — engaging dietitians, other health professionals.<br>Include students' own health behaviors as part of interventions — this personal relevance may improve engagement.<br>Use novel pedagogical approaches, such as culinary medicine (hands-on cooking), to make learning more practical.<br>Develop and use standardized national / international guidance or competency frameworks to define learning objectives and outcomes.<br>Future research should examine long-term impacts, including on physician behavior	Rapid reviews are less exhaustive than full systematic reviews; potential for missing relevant studies.<br>Heterogeneity of interventions makes direct comparison difficult.<br>Many studies used self-reported outcomes (e.g., knowledge or attitudes), which may be subject to bias.<br>Short-term follow-up in many studies; few measured long-term retention or behavior change.
